# Remote CMR 4D Flow Quantification of Pulmonary Flow

**DOI:** 10.1186/1532-429X-18-S1-P307

**Published:** 2016-01-27

**Authors:** Raluca G Saru, Kevin Wanambiro, Albert Hsiao, Laurens E Swart, Sara Boccalini, Mika Vogel, Ricardo Budde, Shreyas Vasanawala, Jolien Roos-Hesselink, Koen Nieman

**Affiliations:** 1 Radiology and Cardiology, Erasmus MC, Rotterdam, Netherlands; 2 University of california, San Diego, CA USA; 3 Stanford University, Palo Alto, CA USA

## Background

4D MR flow has shown to have advantages over standard cardiac magnetic resonance (CMR), offering both anatomical and functional information in just a single acquisition. Pulmonary stenosis and pulmonary regurgitation are common problems in follow-up of patients with congenital heart disease.

In this study we tested flow quantification at the level of the pulmonary valve (forward and backward flow, regurgitation fraction and peak systolic velocity) using a cloud-based software platform fully integrated with correction for eddy currents, Maxwell phase effects and gradient field non-linearity, visualization of the flow and anatomy, and flow quantification. Standard planar phase contrast CMR was used as a reference.

## Methods

Between July 2014 and April 2015, we prospectively included 50 consecutive adult patients planned for CMR with a clinical indication for flow measurement at the level of pulmonary valve and contrast administration. The 4D flow raw data sets were uploaded to a dedicated web-based software application (Arterys Inc., San Francisco, CA, USA).

Images were reconstructed in 20 cardiac temporal phases separately with a compressed sensing algorithm. The forward and backward flow, the regurgitant fraction and the peak systolic velocity measured by CMR 4D flow were compared against planar CMR measurements. To assess the usefulness of corrections for Maxwell phase effects, encoding errors and eddy-currents, these corrections have been turned off and flow calculations were re-done and compared against standard CMR measurements.

## Results

The mean forward flow over the pulmonary valve was 92 ( ± 30) ml/beat for CMR 4D flow and 86 ( ± 30) ml/beat for planar CMR. The Pearson's correlations between CMR 4D flow and CMR were 0.87, 0.95 and 0.85 for forward flow, backward flow and regurgitant fraction respectively. If the corrections for Maxwell phase effects, encoding errors and eddy-currents were not activated for 4D CMR Flow, the correlation for the forward flow was 0.275.

To identify clinically relevant moderate and severe regurgitation, we have used a threshold of 20% of regurgitant fraction. This resulted in sensitivity of 83% (95%CI: 36% - 100%), specificity of 98% (95%CI: 88% - 100%), positive predictive value of 83% (95%CI: 36% - 100%), negative predictive value of 98% (95%CI: 88% - 100%) and accuracy of 96% for CMR 4D flow imaging.

The mean peak systolic velocity measured with CMR 4D flow was 123 ( ± 55) cm/sec and 96 ( ± 51) cm/sec measured with planar CMR and the correlation between the two modalities was 0.78.

## Conclusions

In this study we showed that pulmonary regurgitation can be quantified accurately using CMR 4D flow imaging analysed using a cloud based software. Corrections for Maxwell phase effects, encoding errors and eddy-currents improves overall accuracy of the technique enabling standardized offsite evaluation of CMR examinations.

